# Does dexmedetomidine reduce the risk of acute kidney injury after cardiac surgery? A meta-analysis of randomized controlled trials

**DOI:** 10.1016/j.bjane.2023.07.003

**Published:** 2023-07-14

**Authors:** Chunxiao Zhao, Shuo Liu, Huiquan Zhang, Mengqi Gao

**Affiliations:** a Capital Medical University, Beijing Shijitan Hospital, Department of Intensive Care Unit, Beijing, China; b Capital Medical University, Beijing Chest Hospital, Beijing Tuberculosis and Thoracic Tumor Research Institute, Department of Pharmacy, Beijing, China

**Keywords:** Acute kidney injury, Cardiac surgery, Dexmedetomidine, Meta-analysis

## Abstract

**Background:**

Acute Kidney Injury (AKI) is a common complication after cardiac surgery and has been associated with poor outcomes. Dexmedetomidine (DEX) has been shown to confer direct renoprotection based on some animal and clinical studies, but data from other trials came to the opposite conclusion following cardiac surgery. This meta-analysis was conducted to evaluate the effects of perioperative DEX administration on the occurrence of AKI and the outcomes after cardiac surgery.

**Methods:**

We searched databases including EMBASE, PubMed, and Cochrane CENTRAL for Randomized Controlled Trials (RCTs) focused on DEX for AKI in adult patients after cardiac surgery. The primary outcome was incidence of AKI. Secondary outcomes were Mechanical Ventilation (MV) duration, Intensive Care Unit (ICU) Length Of Stay (LOS), hospital LOS and mortality.

**Results:**

Fifteen trials enrolling 2907 study patients were collected in the meta-analyses. Compared with controls, DEX reduced the incidence of postoperative AKI (Odds Ratio [OR = 0.66]; 95% Confidence Interval [95% CI 0.48–0.91]; *p* = 0.01), and there was no significant difference between groups in postoperative mortality (OR = 0.63; 95% CI 0.32–1.26; *p* = 0.19), MV duration (Weighted Mean Difference [WMD = -0.44]; 95% CI -1.50–0.63; *p* = 0.42), ICU LOS (WMD = -1.19; 95% CI -2.89–0.51; *p* = 0.17), and hospital LOS (WMD = -0.31; 95% CI -0.76–0.15; *p* = 0.19).

**Conclusions:**

Perioperative DEX reduced the incidence of postoperative AKI in adult patients undergoing cardiac surgery. No significant decrease existed in mortality, MV duration, ICU LOS and hospital LOS owing to DEX administration.

## Introduction

Acute Kidney Injury (AKI) is a recognized complication following cardiac surgery with a reported incidence between 5% and 42%.^1^ Postoperative AKI results in poor outcomes, prolonged hospital Length of Stay (LOS), increased hospital costs and mortality.^2^ The mechanism of AKI after cardiac surgery is tightly associated with the hemodynamic instability and sympathetic activity during Cardiopulmonary Bypass (CPB).^3^, ^4^, ^5^ Although numerous trials attempted to identify strategies to prevent AKI, the incidence is still around 40% and no definite strategy exists yet.^6^, ^7^, ^8^, ^9^, ^10^

Dexmedetomidine (DEX) is a highly selective α2 adrenoreceptor agonist and has been widely used for sedation during cardiac surgery. DEX differs from other sedatives by the properties of anti-inflammatory and sympatholytics.^11^^,^^12^ These properties offer a hypothesis that DEX might reduce the incidence of postoperative AKI. Preclinical studies indicated the renoprotective effect of DEX in various animal models.^13^, ^14^, ^15^ Several single-center Randomized Controlled Trials (RCTs) have also addressed this question and the results are controversial.^16^, ^17^, ^18^, ^19^ Previous meta-analyses had evaluated the effect of DEX in cardiac surgery and showed a reduced risk of postoperative AKI.^20^, ^21^, ^22^ However, the studies were limited by high heterogeneity and relatively small sample size. Moreover, some strengthened studies focused on this issue were published in recent years.^23^^,^^24^ Therefore, we conducted this meta-analysis to assess if DEX is associated with a protective effect of AKI after cardiac surgery.

## Methods

### Search strategy and study criteria

This meta-analysis was performed according to the PRISMA (Preferred Reporting Items for Systematic Reviews and Meta-analyses) guidelines^25^ and three electronic databases including MEDLINE (through PubMed), Embase (through OVID) and Cochrane Library were searched to identify relevant studies. The search strategy for PubMed was performed using the keywords “dexmedetomidine”, “cardiac surgery”, “heart surgery”, “kidney”, and “renal”. Various combinations of key words and different search strategies were developed for another two databases. The search encompassed the period between January 1997 and November 2022. All eligible studies met the following conditions: 1) Randomized controlled trials only, and as an original article, 2) Studies published in English, 3) Adult patients undergoing cardiac surgery with or without cardiopulmonary bypass, including coronary artery bypass graft or cardiac valve replacement or coronary artery bypass graft combined with cardiac valve replacement; 4) Intervention: DEX; 5) Comparison: placebo or control (other therapy); 6) Outcome measure: the incidence of postoperative AKI. Exclusion criteria were as follows: retrospective study, observational study, conference abstracts, expert opinion, review articles, case reports, abstracts, editorials, and letters to the editor, animal studies, studies involving pediatric population, and studies lacking clinical outcome data, and failure to contact the authors. Furthermore, the references of relevant studies were also assessed.

### Literature review and data extraction

The literature review and data extraction were independently completed by 2 investigators. In the case of duplicate records pertaining to a single study, we considered the PubMed database to take precedence. Disagreements were handled by discussion to reach consensus. Quality assessment was completed using the Cochrane risk of bias tool: randomization, allocation concealment, blinding, withdrawals and dropouts, and intention-to-treat analysis. Data extraction included characteristics of included studies and patients.

### Postoperative outcomes

The primary end point was incidence of AKI defined based on three definitions, consisting of KDIGO (Kidney Disease: Improving Global Outcome), RIFLE (Risk, Injury, Failure, Loss of kidney function, and End‐stage kidney disease), AKIN (Acute Kidney Injury Network) and undergoing RRT (Renal Replacement Therapy) for new onset of AKI after cardiac surgery. Secondary outcomes included mortality, Mechanical Ventilation (MV) duration, ICU LOS, and hospital LOS.

### Statistical analysis

For dichotomous outcomes (reported with incidence), we calculated the Odds Ratio (OR) with 95% Confidence Interval (95% CI). For continuous outcomes (reported as mean ± standard deviation, median and interquartile range, or median and range), we calculated mean differences for each study according to the statistical method of Hozo et al.^26^ and used weights to pool the estimate (Weighted Mean Difference ‒ WMD) with 95% CI. Random-effect models were used to analyze the data in light of the heterogeneity. Heterogeneity was assessed with Inconsistency statistic (I^2^). Publication bias was assessed by Begg's test, Egger's test and Macaskill test. Meta-regression and subgroup analysis were conducted to explore the potential sources of significant heterogeneity. Sensitivity analyses were used to assess the robustness of our results by removing each included study at one time to obtain and evaluate the remaining overall estimates: *p* < 0.05 (2 sided) was considered to be statistically significant for hypothesis testing. All statistical analyses were performed in REVMAN (version 5.0; Cochrane Collaboration, Oxford, UK) and Stata (version 15.0; StataCorp LP).

## Results

### Study characteristics

**Figure 1:**
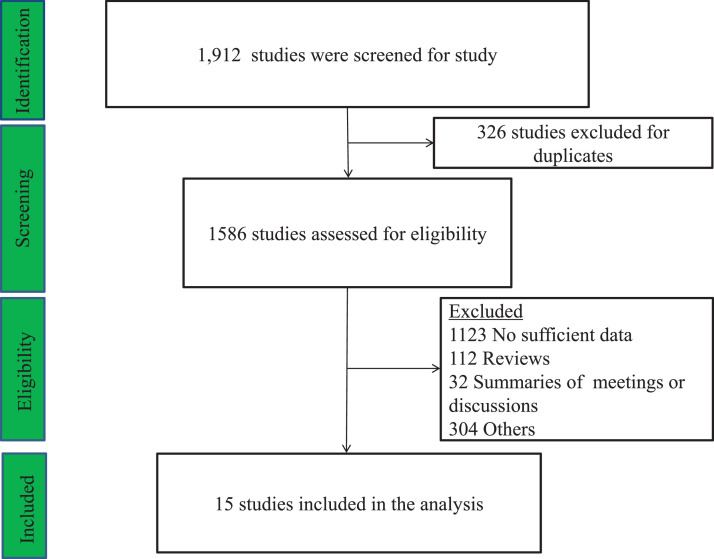
Flow diagram of studies included into meta-analyses.

Figure 1 shows the flow chart for the study screening and selection process in this meta-analysis. Fifteen trials with sixteen groups of data ultimately met our criteria.^16^, ^17^, ^18^, ^19^^,^^23^^,^^24^^,^^27^, ^28^, ^29^, ^30^, ^31^, ^32^, ^33^, ^34^, ^35^ Two studies were for coronary artery bypass grafting, nine were for combined cardiac surgery, two for valve replacement surgery, and two for aortic vascular surgery. Nine trials used placebo as control, whereas four used propofol, one used morphine or remifentanil. DEX was continuously infused at a rate of approximately 0.2 to 0.8 mcg.kg^−1^.h^−1^ for 24 hours after a loading dose (0.4–1 mcg.kg^−1^) in six studies or infused at a rate of approximately 0.04 to 1.5 mcg.kg^−1^.h^−1^ without a loading dose in nine. DEX was used intraoperatively in eleven studies and postoperatively in four.

For primary outcomes, AKI incidence was reported in fifteen trials, including two showing the number of patients needed for dialysis owing to the new onset of AKI after cardiac surgery; For second endpoint, mortality, in seven; mechanical ventilation duration, in twelve; ICU LOS, in thirteen; and hospital LOS, in ten.

**Figure 2:**
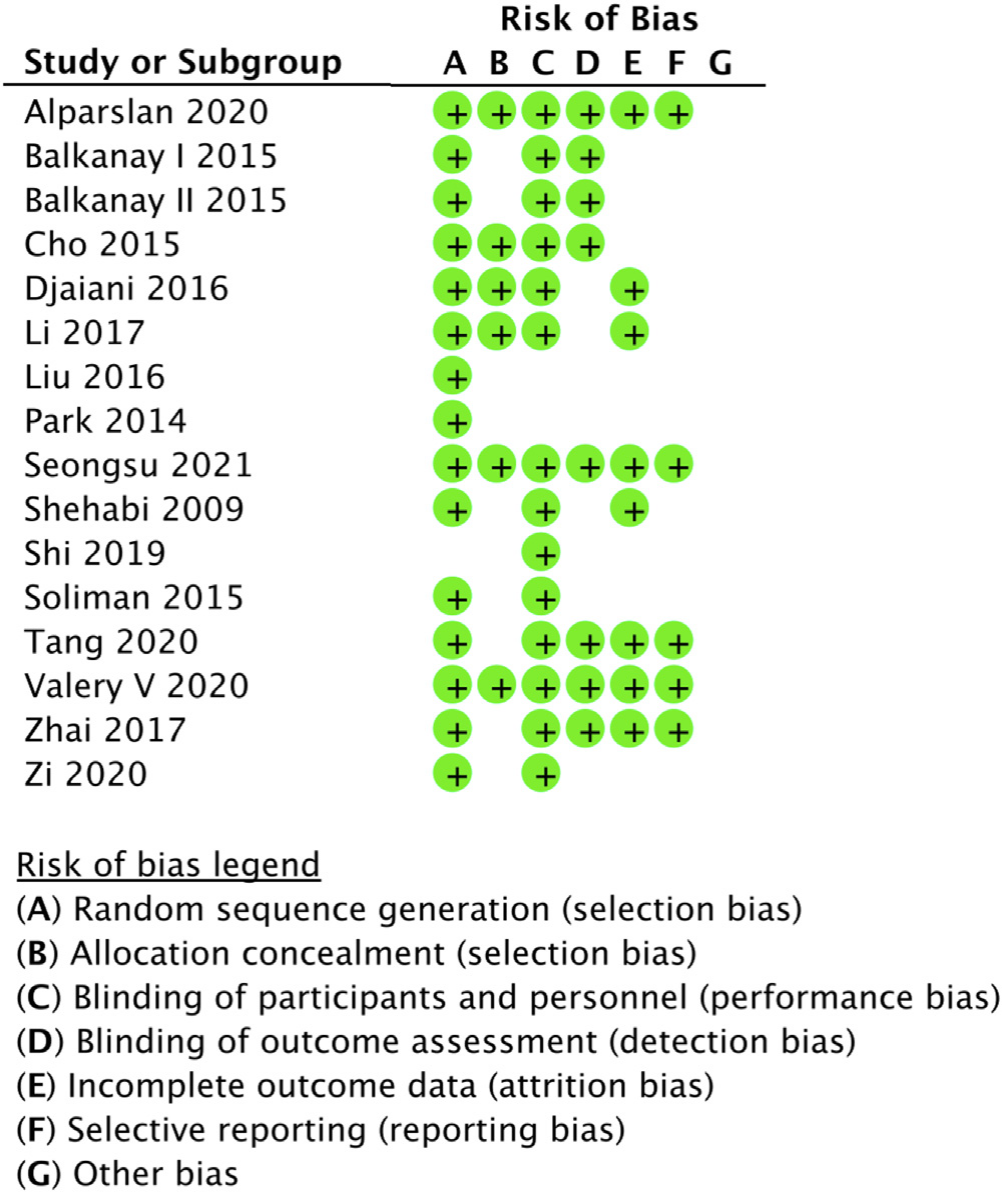
Quality assessment of studies included into meta-analyses.

Study design and patient characteristics are summarized in Tables 1 and 2. The quality assessment is listed in Figure 2 and Table 3.

**Table 1:** Summarized study design of included randomized trials.

Study	Country	Surgery	Dexmedetomidine dose	Control	Time and duration of intervention or control	N° of patients	Clinical end point	AKI definition	Follow-up
Balkanay 2015I	Turkey	On-PUMP CABG	0.04 μg.kg^−1^.h^−1^ ‒0.05 μg.kg^−1^.h^−1^	Placebo	Start preCPB and last for 24h	31 vs. 28	AKI; MV duration; ICU stay; Hospital stay	RIFLE	In hospital
Balkanay 2015II	Turkey	On-PUMP CABG	0.04 μg.kg^−1^.h^−1^ -0.05 μg.kg^−1^.h^−1^	Placebo	Start preCPB and last for 24h	29 vs. 28	AKI; MV duration; ICU stay; Hospital stay	RIFLE	In hospital
Valery 2020	Russia	Combined	0.4 μg.kg^−1^.h^−1^ ‒0.7 μg.kg^−1^.h^−1^	Placebo	Started in the surgical theater and continued in the intensive care unit	84 vs. 85	AKI; Mortality; MV duration; ICU stay; Hospital stay	NA	In hospital
Cho 2015	Korea	Combined	0.04 μg.kg^−1^.h^−1^	Placebo	Start immediately after anesthetic induction and last for 24h	100 vs. 100	AKI; Mortality; ICU stay;	AKIN	In hospital
Tang 2020	China	Valve surgery	1 µg.kg^−1^	Placebo	Start before induction and last for 15 min	38 vs. 37	AKI; MV duration; ICU stay; Hospital stay	KDIGO	In hospital
			0.3 μg.kg^−1^.h^−1^						
DjaianiG 2016	Canada	Combined	0.4 µg.kg^−1^	Propofol	Start postsurgery and last for 24h	91 vs. 92	AKI; Mortality; MV duration; ICU stay; Hospital stay	NA	In hospital
			0.2‒0.7 μg.kg^−1^.h^−1^						
Alparslan 2020	USA	Combined	0.1 μg.kg^−1^.h^−1^‒ 0.4 μg.kg^−1^.h^−1^	Placebo	Started before the surgical incision and last for 24h	398 vs. 396	AF; Stroke; Mortality; ICU stay; Hospital stay	AKIN	90 days after surgery
Li 2017	China	Combined	0.1 μg.kg^−1^.h^−1^ ‒ 0.6 μg.kg^−1^.h^−1^	Placebo	Start preCPB and last until the end of MV	142 vs. 143	AKI; MV duration; ICU stay	KDIGO	30 days after surgery
Liu 2016	China	Combined	< 1.5 μg.kg^−1^.h^−1^	Propofol	Start after surgery and last until the end of MV	44 vs. 44	AKI; Mortality; MV duration; ICU stay; Hospital stay	AKIN	In hospital
Zhai 2017	China	Valve surgery	0.6 µg.kg-^1^	Placebo	before anesthesia and last until the end of operation	36 vs. 36	AKI; MV duration	RIFLE	In hospital
			0.2 μg.kg^−1^.h^−1^						
Park 2014	Korea	Combined	0.5 ug.kg^−1^	Remifentanil	Start after surgery and last until extubation	67 vs. 75	AKI; MV duration; ICU stay; Hospital stay	Cr > 100%abovebaseline or new dialysis need	In hospital
			0.2‒0.8 μg.kg^−1^.h^−1^						
Zi 2020	China	Off-PUMP CABG	0.2‒1 μg.kg^−1^.h^−1^	Propofol	Start from analepsia until the end of ICU	62 vs. 61	AF; MV duration; ICU stay	NA	In hospital
Shehabi 2009	Australia	Combined	0.1‒0.7 µg.kg^−1^.mL^−1^	Morphine	Start within 1h of adminssin to CICU until the removal of chest drains	152 vs. 147	AKI; Mortality; MV duration; ICU stay; Hospital stay	NA	12 days after surgery
Seongsu 2021	Korea	Thoracic aortic surgery	0.4 mg.mL^−1^	Placebo	After the induction until 12h after ACC-off	26 vs. 25	AF; Stroke; MV duration; ICU stay; Hospital stay	NA	In hospital
Shi 2019	China	Combined	0.4–0.6 μg.kg^−1^.h^−1^	Propofol	NA	84 vs. 80	AF; MV duration; ICU stay; Hospital stay	NA	In hospital
Soliman 2016	Egypt	Aortic vascular surgery	1 μg.kg^−1^	Placebo	Start 15 min before induction maintained to the end of surgery	75 vs. 75	AKI; Mortality;	Cr > 115 µmol.L^−1^	In hospital
			0.3 μg.kg^−1^.h^−1^						

AKI, Acute Kidney Injury; CABG, Coronary Artery Bypass Graft; CPB, Cardiopulmonary Bypass; ICU, Intensive Care Unit; CICU, Cardiac Intensive Care Unit, MV, Mechanical Ventilation; NA, Not Available; Cr, Creatinine. RIFLE, Risk-Injury-Failure-Loss-End-stage renal disease; AKIN, Acute Kidney Injury Network; KDIGO, Kidney Disease Improving Global Outcomes.

**Table 2:** Summarized patient characteristics of the included randomized trials.

Study	Age	Male (%)	DM (%)	HP (%)	PreMI (%)	LVEF (%)	CPB duration (min)	Anesthetics	Baseline serum creatinine	β-blocker (%)	Statins (%)
Balkanay 2015I	NA	NA	NA	NA	NA	NA	NA	NA	NA	NA	NA
Balkanay 2015II	NA	NA	NA	NA	NA	NA	NA	NA	NA	NA	NA
Valery 2020	62.5	72.2	43.8	79.3	27.8	55.4	121.5	Sevoflurane	NA	63.9	NA
Cho 2015	63	48	19.5	45.5	NA	61.5	131	Sevoflurane	33	NA	63
Tang 2020	55.0	61.3	NA	NA	NA	57.9	71.0	Sevoflurane	NA	NA	NA
DjaianiG 2016	72.55	75.4	21.9	75.4	16.4	NA	98.99	Isoflurane	53	68.85	72.55
Alparslan 2020	62.5	69.6	20.8	67.1	10.8	60	NA	NA	NA	49.1	55
Li 2017	67.18	69.1	32.3	63.2	9.8	NA	102.99	Sevoflurane	69.73	48.42	67.18
Liu 2016	54.75	39.8	12.5	29.5	NA	65	71.15	Sevoflurane	NA	NA	54.75
Zhai 2017	46	45.8	NA	NA	NA	49	72.5	NA	NA	NA	NA
Park 2014	53.81	55.6	9.15	27.5	NA	61.87	166.75	Sevoflurane	NA	NA	53.81
Zi 2020	65.4	67.5	46.3	64.2	16.3	56.5	NA	NA	NA	NA	
Shehabi 2009	71.25	75.3	29.5	80.1	36.6	NA	98.98	Sevoflurane	NA	NA	71.25
Seongsu 2021	61.5	54.9	11.8	68.6	13.7	63	NA	NA		23.5	NA
Shi 2019	74.5	72.6	NA	NA	NA	NA	112.9	NA		54.3	79.9
Soliman 2016	58.1	50	30.7	48.7	8.6	52.9	NA	NA	36.67	NA	58.1

Note: Values are given as means unless otherwise specified.

DM, Diabetes Mellitus; HP, Hypertension; PreMI, Previous Myocardial Infarction; LVEF, Left Ventricular Ejection Fraction; CPB, Cardiopulmonary Bypass; NA, Not Available.

**Table 3:** Summarized quality assessment of included randomized trials.

Study	Random sequencegeneration	Allocation Concealment	Blinding of participants and personnel	Blinding of outcome assessment	Attrition bias	Selective reporting
Balkanay 2015I	Low risk	Unclear	Low risk	Low risk	Unclear	Unclear
Balkanay 2015II	Low risk	Unclear	Low risk	Low risk	Unclear	Unclear
Valery 2020	Low risk	Low risk	Low risk	Low risk	Low risk	Low risk
Cho 2015	Low risk	Low risk	Low risk	Low risk	Unclear	Unclear
Tang 2020	Low risk	Unclear	Low risk	Low risk	Low risk	Low risk
Djaiani G 2016	Low risk	Low risk	Low risk	Unclear	Low risk	Unclear
Alparslan 2020	Low risk	Low risk	Low risk	Low risk	Low risk	Low risk
Li 2017	Low risk	Low risk	Low risk	Unclear	Low risk	Unclear
Liu 2016	Low risk	Unclear	Unclear	Unclear	Unclear	Unclear personnel
Zhai 2017	Low risk	Unclear	Low risk	Low risk	Low risk	Low risk
Park 2014	Low risk	Unclear	Unclear	Unclear	Unclear	Unclear
Zi 2020	Low risk	Unclear	Low risk	Unclear	Unclear	Unclear
Shehabi 2009	Low risk	Unclear	Low risk	Unclear	Low risk	Unclear
Seongsu 2021	Low risk	Low risk	Low risk	Low risk	Low risk	Low risk
Shi 2019	Unclear	Unclear	Low risk	Unclear	Unclear	Unclear
Soliman 2016	Low risk	Unclear	Low risk	Unclear	Unclear	Unclear

### Effect of DEX on incidence of AKI, and mortality

**Figure 3:**
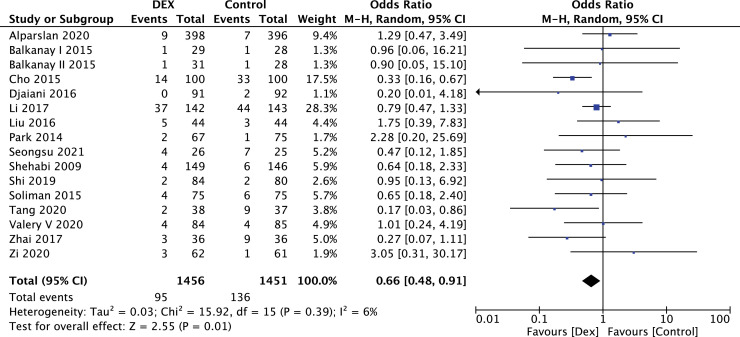
DEX reduced the incidence of AKI.

The outcome of AKI was reported in 2907 study participants, and the overall incidence was 7.95% (DEX group, 6.52%; control group, 9.37%). The postoperative incidence of AKI was significantly reduced by DEX (fifteen studies; OR = 0.66; 95% CI 0.48–0.91; *p* = 0.01; I^2^ = 6%); (Fig. 3). There was no evidence of publication bias (Begg's test *p* = 0.96; Egger's test *p* = 0.55).

Subgroup analyses revealed similar trends to those of postoperative AKI outcome based on different characteristics such as age (≥ 62.5 vs. < 62.5 years), male proportion (≥ 62% vs. < 62%), diabetes proportion (≥ 25% vs. < 25%), hypertension proportion (≥ 25% vs. < 25%), previous Myocardial Infarction (MI) proportion (≥ 15% vs. < 15%), Left Ventricular Ejection Fraction (LVEF) (≥ 60% vs. < 60%), Cardiopulmonary Bypass (CPB) duration (≥ 100 vs. < 100 min), β-blocker (≥ 50% vs. < 50%), Statin (≥ 65% vs. < 65%), loading dose (use or not), type of control (placebo vs. others), administration timing (pre/intraoperative vs. postoperative) and surgery type (combined surgery vs. others) (Supplementary Table 1).

Meta-regression analyses performed for the potential sources of significant heterogeneity are listed in Supplementary Table 2, and there were no significant differences for postoperative AKI in all the subgroups.

Sensitivity analyses excluding each included study at a time revealed that all the studies were consistent with the direction and size of the overall AKI-reducing effect of DEX (*p* < 0.05 for all) except Cho.

The outcome mortality was reported in 1883 study participants, and the overall incidence was 1.86% (DEX group, 1.38%; control group, 2.34%). There was no significant difference between DEX and the risk of mortality (Seven studies; OR = 0.63; 95% CI 0.32–1.26; *p* = 0.19; I^2^ = 0%); (Supplementary Figure 1).

### Effect of DEX on MV duration, ICU LOS, and hospital LOS

Postoperative MV duration was reported in twelve studies, and no statistically significant reduction by DEX was found (eleven studies; WMD = -0.44; 95% CI -1.50–0.63; *p* = 0.42; I^2^ = 73%); (Supplementary Fig. 2). There was no significant difference in ICU LOS (thirteen studies; WMD = -1.19; 95% CI -2.89–0.51; *p* = 0.17; I^2^=74%); (Supplementary Fig. 3), as well as in hospital LOS (ten studies; WMD = -0.31; 95% CI -0.76–0.15; *p* = 0.19; I^2^ = 76%); (Supplementary Fig. 4).

## Discussion

In this meta-analysis of fifteen RCTs involving 2907 adult patients undergoing cardiac surgery, we found that perioperative DEX was associated with a decrease in postoperative AKI. However, postoperative parameters including MV duration, ICU, hospital LOS and mortality did not seem to present a significant reduction as a result of the DEX.

AKI is common after cardiac surgery and small increases in postoperative serum creatinine levels have been reported to be related with worse outcome, even when renal function returns to normal ultimately.^36^^,^^37^ The reason that cardiac surgery can cause AKI is always accompanied by renal Ischemia-Reperfusion Injury (I/RI), elevated sympathetic activity, and hemodynamic instability. For this reason, pharmacologic or other prophylaxis which have these properties may reduce AKI after cardiac surgery and this is an important research area to clinicians.^38^, ^39^, ^40^

DEX has been widely used in anesthesia procedures and has shown organ protection by stabilizing the sympathetic system, exerting anti-inflammatory effects, and attenuating Ischemia/Reperfusion (I/R) injury in vivo and vitro studies.^41^, ^42^, ^43^, ^44^, ^45^ There is a hypothesis that the incidence of AKI may be reduced owing to the use of DEX in cardiac surgery.^46^^,^^47^ Several studies have compared the efficacy of DEX at enhancing urine output and at decreasing the concentration of blood urea nitrogen and creatinine after surgery,^19^^,^^48^^,^^49^ and other randomized controlled trials have reported a lower rate of kidney injury.^17^^,^^50^^,^^51^ No general consensus was reached on the effect of DEX for AKI.^52^, ^53^, ^54^ A few meta-analyses have been conducted to address this issue. However, a meta-analysis performed by Peng,^20^ which included nine RCTs with a total of 1308 patients, showed low heterogeneity (I^2^ = 30%). Another meta-analysis by Liu[21] including ten RCTs with a total of 1575 patients showed only eight groups of data from seven studies on the main outcome. Our study with an almost two times larger sample size collected some high-quality research published in recent years and provided a more convincing conclusion.

Based on our literature review, positive reno-protective effects were reported in two studies. Moreover, in our data analysis, the combined results with a random-effects model revealed lower AKI incidence in patients with DEX, and the pooled OR succeeded to reach statistical significance. However, this benefit did not translate into the second outcomes, such as MV duration, ICU LOS, hospital LOS and mortality. A possible explanation is that our meta-analysis with a relatively small sample size may account for such differences. Another is that heterogeneity for the MV duration, ICU and hospital LOS is almost over 50%. In fact, there are trends toward lower MV duration, ICU LOS, hospital LOS and mortality. Further randomized studies with large sample sizes are encouraged to verify the current findings.

Our analysis has several limitations. Firstly, many factors could influence AKI after cardiac surgery, such as age, degree of hypertension, and drugs used for treating hypertension and diabetes mellitus. We were unable to access individual patient data, so the influences of confounding factors may be underestimated. Secondly, we only included English language trials and published studies, which may lead to publication bias. Thirdly, many design differences among these studies made it difficult to reduce clinical heterogeneity. Subgroup analyses and meta-regression were performed for the potential sources of heterogeneity. Finally, based on the included data, there are four different definitions of AKI, including RIFLE, AKIN, KDIGO, and need for RRT. Six studies did not mention the definition of AKI. According to previous studies,^55^^,^^56^ the incidence of AKI can vary greatly according to the definition used, and our study might draw a misleading conclusion. Given only three or less studies were included, a subgroup analysis based on AKI definition was not performed.

## Conclusion

In summary, our meta-analysis indicated that perioperative DEX use reduced postoperative AKI in patients receiving cardiac surgery. However, DEX use is not associated with MV duration, ICU LOS, hospital LOS and mortality. Future, much larger trials are needed to verify the current findings.

## Data availability statement

The data used to support the findings of this study are included within the supplementary information file.

## Funding statement

This research received no specific grant from any funding agency in the public, commercial, or not-for-profit sectors.

## Ethical statement

Since this was a meta-analysis, ethical approval was not required under the arrangements of the Institutional Review Board in our hospital.

## Declaration of Competing Interest

The authors declare no conflicts of interest.

## Appendix Supplementary materials

Image, application 1

## Footnotes

Supplementary material associated with this article can be found in the online version at doi:10.1016/j.bjane.2023.07.003.
