# Supplementary material for: Does dexmedetomidine reduce the risk of acute kidney injury after cardiac surgery? A meta-analysis of randomized controlled trials
Source: Braz J Anesthesiol. 2023 Jul 14;74(3):744446. doi: 10.1016/j.bjane.2023.07.003 (PMC11148486; doi:10.1016/j.bjane.2023.07.003)
Supplement: Supplementary file 1 [file mmc1.docx]

**BJAN-D-23-00022_Supplementary Material**

**Supplementary Figure 1** Forest plot for postoperative mortality.

**Supplementary Figure 2** Forest plot for MV duration.

**Supplementary Figure 3** Forest plot for ICU LOS.

**Supplementary Figure 4** Forest plot for hospital LOS.

**Supplementary Table 1** Subgroup analyses for the potential sources of heterogeneity.

| **Subgroup** | **Endpoint** | **Nº of Comparisons** | **OR WMD** | **95% CI** | **p-value** | **I^2^** | **p^Difference^ value** |
| --- | --- | --- | --- | --- | --- | --- | --- |
| Age (years) | AKI | 14 | 0.65 | 0.45~ 0.94 | 0.02 | 18% | 0.91 |
| ≥ 62.5 |  | 7 | 0.64 | 0.41~ 0.99 | 0.05 | 14% |  |
| < 62.5 |  | 7 | 0.67 | 0.35~ 1.28 | 0.19 | 32% |  |
| Gender (male%) | AKI | 14 | 0.65 | 0.45~ 0.94 | 0.02 | 18% | 0.91 |
| ≥ 62 |  | 7 | 0.88 | 0.59~ 1.30 | 0.51 | 0% |  |
| < 62 |  | 7 | 0.47 | 0.26~ 0.82 | 0.008 | 23% |  |
| DM (%) | AKI | 11 | 0.73 | 0.51~ 1.04 | 0.08 | 11% | 0.75 |
| ≥ 25 |  | 5 | 0.81 | 0.53~ 1.24 | 0.33 | 0% |  |
| < 25 |  | 6 | 0.71 | 0.34~ 1.48 | 0.36 | 43% |  |
| HP (%) | AKI | 11 | 0.73 | 0.51~ 1.04 | 0.08 | 11% | 0.88 |
| ≥ 25 |  | 5 | 0.81 | 0.44~ 1.48 | 0.49 | 0% |  |
|  |  |  |  | 0.34~ 1.48 |  |  |  |
| < 25 |  | 6 | 0.76 | 0.42~ 1.38 | 0.36 | 41% |  |
| Previous MI (%) | AKI | 8 | 0.81 | 0.56 ~ 1.18 | 0.28 | 0% | 0.90 |
| ≥ 15 |  | 4 | 0.85 | 0.37 ~ 1.99 | 0.71 | 0% |  |
| < 15 |  | 4 | 0.81 | 0.53 ~ 1.22 | 0.30 | 0% |  |
| LVEF (%) | AKI | 10 | 0.65 | 0.38 ~ 1.11 | 0.11 | 37% | 0.55 |
| ≥ 60 |  | 5 | 0.77 | 0.35 ~ 1.70 | 0.52 | 52% |  |
| < 60 |  | 5 | 0.54 | 0.24 ~ 1.25 | 0.15 | 32% |  |
| CPB duration (minutes) | AKI | 10 | 0.58 | 0.37 ~ 0.91 | 0.02 | 24% | 0.50 |
| ≥ 100 |  | 5 | 0.65 | 0.38 ~ 1.13 | 0.13 | 30% |  |
| < 100 |  | 5 | 0.46 | 0.20 ~ 1.08 | 0.07 | 28% |  |
| β-blocker (%) | AKI | 6 | 0.82 | 0.55 ~ 1.23 | 0.34 | 0% | 0.98 |
| ≥ 50 |  | 3 | 0.81 | 0.28 ~ 2.38 | 0.70 | 0% |  |
| < 50 |  | 3 | 0.82 | 0.53 ~ 1.27 | 0.38 | 0% |  |
| Statin (%) | AKI | 9 | 0.82 | 0.55 ~ 1.23 | 0.34 | 0% | 0.98 |
| ≥ 65 |  | 4 | 0.81 | 0.28~ 2.38 | 0.70 | 0% |  |
| < 65 |  | 5 | 0.82 | 0.53 ~ 1.27 | 0.49 | 0% |  |
| Loading dose use | AKI | 15 | 0.66 | 0.48 ~ 0.91 | 0.01 | 6% | 0.12 |
| Yes |  | 6 | 0.41 | 0.21 ~ 0.80 | 0.009 | 0% |  |
| No |  | 9 | 0.75 | 0.53 ~ 1.07 | 0.11 | 4% |  |
| Control drugs | AKI | 15 | 0.66 | 0.48 ~ 0.91 | 0.01 | 6% | 0.15 |
| Placebo |  | 9 | 0.59 | 0.40 ~ 0.86 | 0.006 | 14% |  |
| Others |  | 6 | 1.09 | 0.51 ~ 2.31 | 0.83 | 0% |  |
| Dex administration | AKI | 14 | 0.65 | 0.46 ~ 0.92 | 0.02 | 11% | 0.16 |
| Pre/Intraoperation |  | 9 | 0.59 | 0.40 ~ 0.86 | 0.006 | 14% |  |
| Postoperation |  | 5 | 1.11 | 0.49 ~ 2.51 | 0.80 | 0% |  |
| Surgical procedures | AKI | 15 | 0.66 | 0.48 ~ 0.91 | 0.01 | 6% | 0.26 |
| CABG or Aortic surgery |  | 6 | 0.48 | 0.25 ~ 0.91 | 0.02 | 0% |  |
| Combined |  | 9 | 0.74 | 0.49 ~ 1.10 | 0.14 | 15% |  |

AKI, Acute Kidney Injury; OR, Odds Ratio; CI, Confidence Interval; DM, Diabetes Mellitus; CPB, Cardiopulmonary Bypass; Dex, Dexmedetomidine; CABG, Coronary Artery Bypass Graft.

**Supplementary Table 2** Meta-regression for the potential sources of heterogeneity.

|  | **Regression coefficient** | **95% CI** | **p-value** |
| --- | --- | --- | --- |
| **Age (years)** | 0.022 | -0.035~ 0.080 | 0.456 |
| **Gender (male%)** | 0.023 | -0.004~0.050 | 0.101 |
| **Previous DM (%)** | 0.013 | -0.028~0.056 | 0.531 |
| **CPB duration (minutes)** | 0.006 | -0.015~0.027 | 0.566 |
| **HP (%)** | -0.001 | -0.030~0.028 | 0.938 |
| **PreMI** | -0.004 | -0.048~ 0.041 | 0.878 |
| **LVEF** | 0.046 | -0.074~0.165 | 0.456 |
| **Propofol** | 0.360 | -0.123~0.719 | 0.131 |
| **Betablockers** | 0.013 | -0.031~0.058 | 0.574 |
| **Statins** | -0.033 | -0.101~0.034 | 0.331 |
| **Loading dose use** | 0.021 | -0.002~0.043 | 0.106 |
| **Time of DEX administration (Pre/Intraoperation)** | -0.131 | -0.561~0.332 | 0.512 |
| **Surgery type** | -0.434 | -0.812 ~-0.324 | 0.161 |
| **Jadad score** | 0.146 | -0.341 ~ 0.356 | 0.330 |

CI, Confidence Interval; DM, Diabetes Mellitus; CPB, Cardiopulmonary Bypass; HP, Hypertension; PreMI, Previous Myocardial infarction; DEX, Dexmedetomidine; LVEF, Left Ventricular Ejection Fraction.
